# A Decade in Focus: Examining Lessons Learned From Office-Directed Injectables in an Academic Practice

**DOI:** 10.1093/asjof/ojaf013

**Published:** 2025-06-30

**Authors:** Vidhya Nadarajan, Bhavana Thota, Anca Dogaroiu, Lauren Kim, Amor Niksic, Victoria Peters, Tuong-Vi Cindy Ngo, Jennifer Barillas, Jeffrey Kenkel

## Abstract

**Background:**

Noninvasive facial rejuvenation procedures have continued to grow in popularity, with botulinum toxin and injectable soft-tissue fillers being the most common.

**Objectives:**

The purpose of this study was to evaluate trends in patient cost, provider product preferences, and complications of a single surgeon's 10-year experience with botulinum toxin and soft-tissue fillers. Additionally, this study aimed to quantify the crossover between patients receiving injectables and patients receiving aesthetic surgery.

**Methods:**

This was a retrospective analysis utilizing internal/departmental records and Epic charting from January 2013 to January 2023. Botulinum toxin and soft-tissue filler cases were captured using select CPT codes, and patient demographics, complications, and follow-up data were recorded.

**Results:**

One thousand three hundred and sixty-eight patients undergoing 5794 injectable cases were assessed. The study population was majority female (89.3%) and Caucasian (78.7%). Injectable cases increased over time, except for a decrease in 2020. Botulinum toxin was most common (59%), followed by fillers (18%), and combination therapy (19%). Common injection sites included the glabella, crow's feet, and forehead for neurotoxins and marionette lines and nasolabial folds for fillers. Complications were rare, with 35 from botulinum toxin and 33 from fillers. Of patients receiving injectables, 19.6% went on to receive an aesthetic surgery, and 0.6% of patients undergoing aesthetic surgery subsequently received injectables.

**Conclusions:**

Botulinum toxin and soft-tissue fillers are safe with low complication rates when administered by an experienced provider. These procedures may serve as a starting point for patients pursuing antiaging treatments and an opportunity to establish continuity of care for providers.

**Level of Evidence: 4 (Therapeutic):**

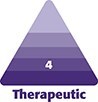

Over the past decade, noninvasive facial rejuvenation procedures have become increasingly popular in the management of the aging face.^1,2^ These procedures offer the benefit of decreased downtime compared with surgery and often provide a suitable alternative for patients who are not ideal surgical candidates or who desire a more conservative result.^3,4^ According to the Aesthetic Society's 2022 Aesthetic Plastic Surgery National Databank Statistics, neurotoxins and dermal fillers were listed as the first and third most common nonsurgical cosmetic procedures, respectively.^5^

Injectable fillers are materials injected in or beneath the skin layers to restore facial volume, smooth wrinkles, and enhance facial contours.^6^ There are 2 categories of injectable fillers—dermal fillers, which physically add volume, and bio-stimulatory fillers, which stimulate the synthesis of new collagen.^7^ Although a wide variety of soft-tissue fillers are available for use, each with their own unique purposes and indications, hyaluronic acid–based fillers are most common.^6,7^

Botulinum toxin Type A (BoNT-A) is a purified neurotoxin produced by the bacterium *Clostridium botulinum*.^8^ It is approved by the FDA to treat a variety of conditions, including muscle spasms, hyperhidrosis, migraines, and moderate-to-severe glabellar lines.^9,10^ Because its approval by the FDA in 2002 for cosmetic use to improve glabellar lines, the use of onabotulinumtoxinA has grown significantly and, over the years, other companies have started to manufacture BoNT-A, such as AbobotulinumtoxinA (Dysport, Galderma, Lausanne, Switzerland), Incobotulinum Toxin (Xeomin, Merz Pharmaceuticals, LLC, Frankfurt am Main, Germany), and DaxibotulinumtoxinA-lanm (Daxxify, Revance Aesthetics, Nashville, TN) in 2009, 2011, and 2022 respectively.^8-12^

Some have hypothesized that neuromodulator injections and soft-tissue fillers may serve as a gateway to cosmetic medicine and ultimately result in these patients undergoing additional antiaging therapies, including surgery, in the future.^13^ A study conducted by Richards et al showed that a significant number of patients who initially sought botulinum toxin and/or soft-tissue filler injections ultimately underwent cosmetic surgery.^14^ Furthermore, previous research found that 47% of patients would choose a nonplastic surgeon for an invasive procedure if they had a prior positive experience with that same provider for a noninvasive procedure.^15^ These studies suggest that noninvasive cosmetic procedures are a crucial element of patient retention in a plastic surgery practice.

The goal of this study is to evaluate trends in patient cost, provider product preferences, and complications of a single surgeon's 10-year experience with neuromodulator injections and soft-tissue fillers. We also share insights into the crossover between patients receiving injectables and patients receiving aesthetic surgical procedures.

## METHODS

### Study Population and Design

**Figure 1.**
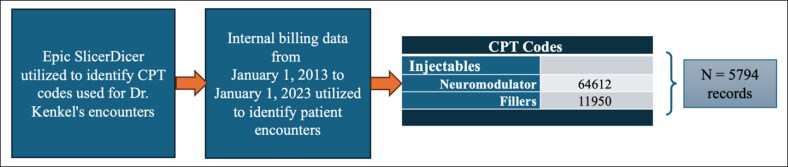
Flow diagram depicting study methodology.

This was a single, high-volume institution retrospective study of all patients age ≥18 undergoing botulinum toxin and/or soft-tissue filler injection procedures performed by the senior author (J.K.) between January 2013 and January 2023. The study protocol was approved by the Institutional Review Board of the study institution. A total of 1368 patients undergoing 5794 procedures were identified using EPIC SlicerDicer (an electronic medical record [EMR] querying tool) and select billing CPT codes (64,612 for chemodenervation and 11,950 for soft-tissue fillers) from departmental records (Figure 1).

### Data Collection and Statistical Analysis

Demographic and procedure outcome data were recorded for all patients in this study. The preprocedural diagnosis, injectable type, volume administered, injection location, procedure cost, history of aesthetic procedures, and dates of all procedures performed were also collected. The primary endpoints of this study were the number of patients who received surgery after first receiving injectables and the number of patients who received injectables after first receiving surgery. Additional endpoints collected included neuromodulator and soft-tissue filler complications and injection sites. Descriptive statistics were performed using Microsoft Excel (Redmond, WA; version 16.77).

## RESULTS

From January 2013 to January 2023, a total of 1368 patients underwent 5614 injectables cases performed by the senior author (J.K.). Patients were mostly female (89.3%, *n* = 1221) and Caucasian (78.7%, *n* = 1076; Table 1).

**Table 1.** Patient Demographics

*Characteristic*	*%*
***Gender***	
*Female*	89.3
*Male*	10.7
***Race***	
*White*	78.7
*Asian*	10.7
*Black*	5.3
*Other*	5.3

**Figure 2.**
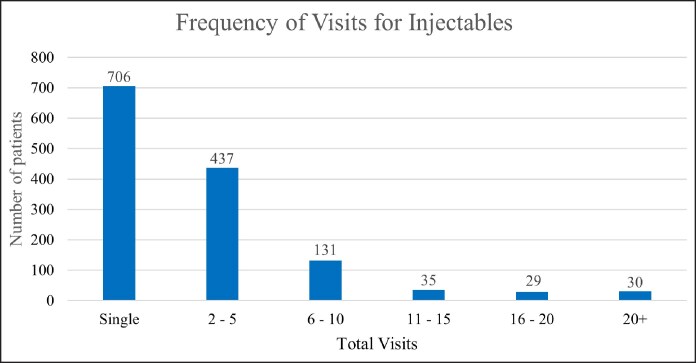
Frequency of injectables visits (number of patients).

The mean visits per patient were 3.4 ± 5, the median number of visits were 1.0, and approximately half (51.6%) of the patients had single visits (*n* = 706). The average age at first encounter for single-visit patients was 53.4 ± 13.1 (17.4-85.7) years and 52.5 ± 12.9 (16.5-84.5) years for multiple-visit patients (Table 2). Of the patients who had multiple visits, 31.9% had 2 to 5 visits (*n* = 437) and 2.2% (*n* = 30) had over 20 visits (Figure 2).

**Table 2.** Patient Visit Details

*Visit History*	*Mean ± SD (min-max) in years*
*Single Visits*	
*First encounter*	53.4 ± 13.1 (17.4 - 85.7)
*Multiple Visits*	
*Most recent encounter*	55.5 ± 13.5 (16.7 - 89.7)
*First encounter*	52.5 ± 12.9 (16.5 - 84.5)
*Time between first and most recent encounter*	2.98 ± 2.88 (.01 - 10.84)

Patient expenditure on injectables over the 10-year period ranged from 48.75 United States dollars (USD) to 50,422 USD, with the majority of patients (*n* = 643, 47%) spending between 0 and 1000 USD (Table 3). The individual overall investment for the 10 patients with the most visits (range, 36-70 visits) is listed in Table 4. Four of these patients also had cosmetic surgery.

**Table 3.** Total Amount ($) Spent on Injectables (2013-2023)

Amount spent	*n*	%
0-1000	643	47.0
1000-2000	281	20.5
2000-3000	147	10.7
3000-5000	114	8.3
5100-10,000	102	7.5
10,000-50,000	80	5.8
>50,000	1	0.1

**Table 4.** Top Investments in Injectables

Patient	Total spent ($)	Visits
1	50,422	70
2	27,111	43
3	24,980	40
4^a^	24,832	39
5^a^	24,134	37
6	23,934	37
7	23,789	37
8^a^	23,485	36
9	22,647	36
10^a^	22,251	36
Total	267,584	411

^a^Patients with surgical procedure.

**Figure 3.**
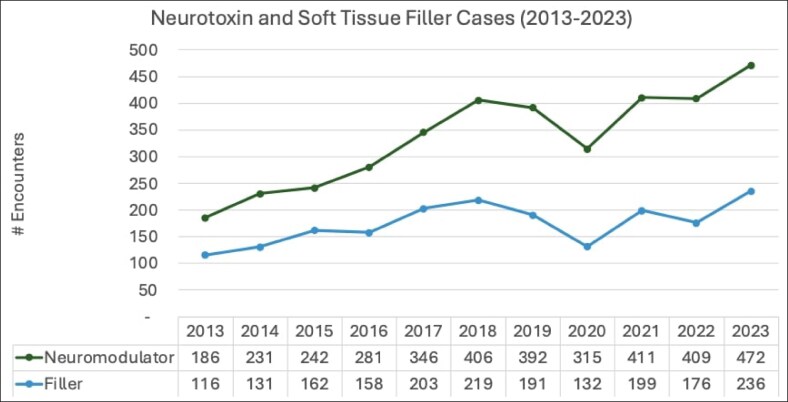
Neurotoxin and soft-tissue filler cases (2013-2023).

**Figure 4.**
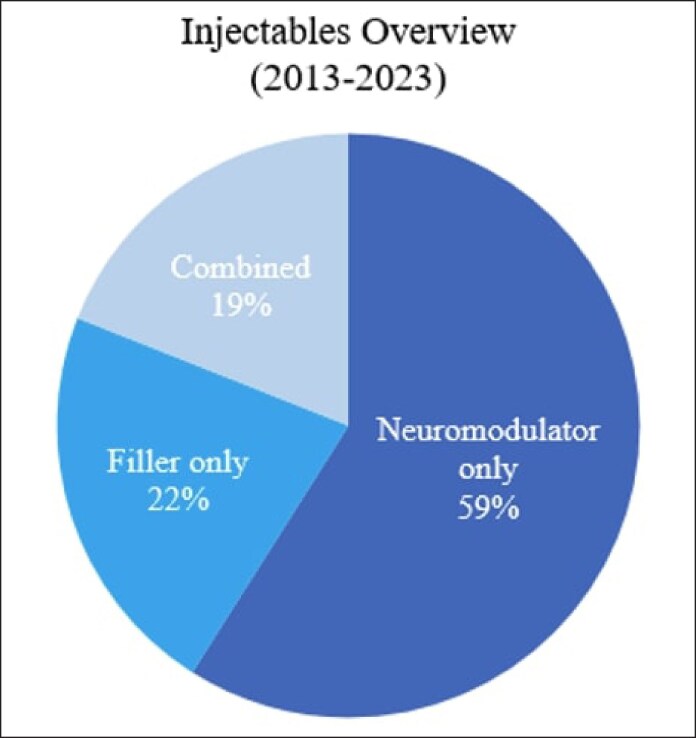
Injectable cases overview.

The number of injectable cases steadily increased over the 10-year period with a drop in 2020 that rapidly corrected (Figure 3). Most cases were botulinum toxin injections only (59%, *n* = 2796), followed by filler only (22%, *n* = 1028) and combination therapy (19%, *n* = 895; Figure 4).

The most frequently used products were Botox (Allergan, Dublin, Ireland)/onabotulinumtoxinA (87.7%, *n* = 3238), Juvéderm (Allergan, Dublin, Ireland)/hyaluronic acid filler (61.9%, *n* = 1310), followed by Restylane (Galderma, Lausanne, Switzerland)/hyaluronic acid filler (28.9%, *n* = 612; Tables 5, 6).

**Table 5.** Injectable Products Overview

Brand name	Product	Frequency	%
Botox (Allergan, Dublin, Ireland)	OnabotulinumtoxinA	3238	87.66
Dysport (Galderma, Lausanne, Switzerland)	AbobotulinumtoxinA	387	10.48
Daxxify (Revance Aesthetics,Nashville, TN)	DaxibotulinumtoxinA-lanm	37	1.00
Hylenex (Halozyme, Inc, San Diego, CA)	Hyaluronidase, Human Recomb.	13	0.35
Kybella (Abbvie, North Chicago, IL)	Deoxycholic acid	10	0.27
Xeomin (Merz, Raleigh, NC)	IncobotulinumtoxinA	8	0.22
Vitrase (Bausch & Lomb Incorporated, Bridgewater, NJ)	Hyaluronidase, Ovine	1	0.03

**Table 6.** Dermal Filler Product Overview

Brand name	Product	Frequency	%
Juvéderm (Allergan, Dublin, Ireland)		1310	61.91
	Ultra	645	30.48
	Voluma	234	11.06
	Vollure	198	9.36
	Ultra Plus	125	5.91
	Volbella	61	2.88
	Not listed	47	2.22
Restylane (Galderma, Lausanne, Switzerland)		612	28.92
	Lyft	183	8.65
	Silk	123	5.81
	Restylane-L	103	4.87
	Defyne	93	4.40
	Refyne	38	1.80
	Kysse	36	1.70
	Perlane	32	1.51
	Contour	4	0.19
Belotero (Merz, Raleigh, NC)	Balance	93	4.40
Revance (Nashville, TN)		70	3.31
	RHA2	26	1.23
	RHA3	23	1.09
	Redensity	17	0.80
	RHA4	4	0.19
Radiesse (Merz, Raleigh, NC)	Calcium hydroxylapatite	21	1.00
Sculptra (Galderma, Lausanne, Switzerland)	Poly-L-lactic acid	10	0.47

**Figure 5.**
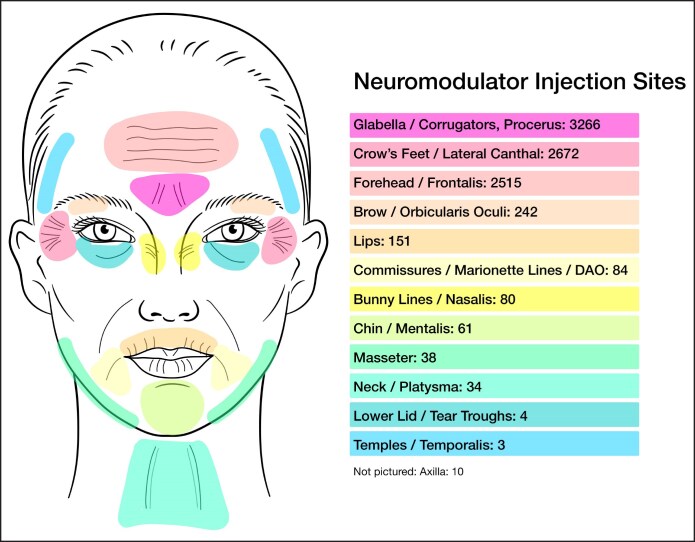
Neuromodulator injection sites.

**Figure 6.**
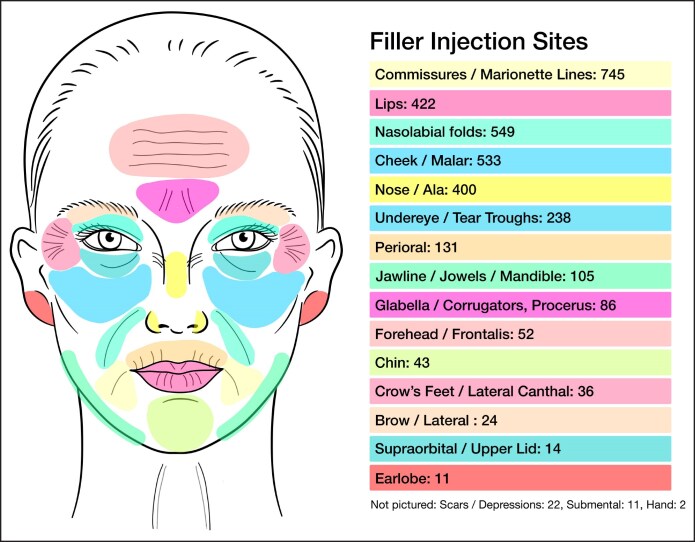
Soft-tissue filler injection sites.

Neuromodulator was most frequently injected into the glabella (corrugators, procerus; *n* = 3266), crow's feet (lateral canthus; *n* = 2672), and forehead (frontalis; *n* = 2515; Figure 5). Soft-tissue filler injection sites included marionette lines/commissures (*n* = 745), nasolabial folds (*n* = 549), midface (*n* = 533), lips (*n* = 422), and nose/ala (*n* = 400; Figure 6).

Of the 3691 total botulinum toxin cases, there were 35 complications/adverse events captured by chart review. These included bruising/ecchymosis (*n* = 10), overactivity/animation (*n* = 7), brow ptosis (*n* = 3), eyelid ptosis (*n* = 3), asymmetry (*n* = 6), edema/swelling (*n* = 2), epiphora (*n* = 1), stye (*n* = 1), cyst (*n* = 1), and recurrence of hyperhidrosis (*n* = 1; Table 7). Of the 1923 total soft-tissue filler cases, there were 33 complications/adverse events captured by chart review. These included edema/swelling (*n* = 9), nodules (*n* = 4), asymmetry (*n* = 4), bruising (*n* = 3), palpability (*n* = 3), infection (*n* = 3), skin depression (*n* = 2), pruritis/itching (*n* = 2), discoloration (*n* = 1), rash (*n* = 1), and erythema/redness (*n* = 1; Table 8).

**Table 7.** Neurotoxin Adverse Events

Adverse event	*n*	%
Bruising/ecchymosis	10	28.57
Overactivity/animation	7	20.00
Brow ptosis	3	8.57
Eyelid ptosis	3	8.57
Asymmetry	6	17.14
Edema/swelling	2	5.71
Epiphora	1	2.86
Stye	1	2.86
Recurrence of hyperhidrosis	1	2.86
Cyst	1	2.86

**Table 8.** Soft-Tissue Filler Adverse Events

Adverse event	*n*	%
Swelling	9	27.27
Nodules	4	12.12
Asymmetry	4	12.12
Bruising	3	9.09
Palpability	3	9.09
Infection	3	9.09
Depression	2	6.06
Itching/pruritus	2	6.06
Discoloration	1	3.03
Rash	1	3.03
Redness	1	3.03

**Figure 7.**
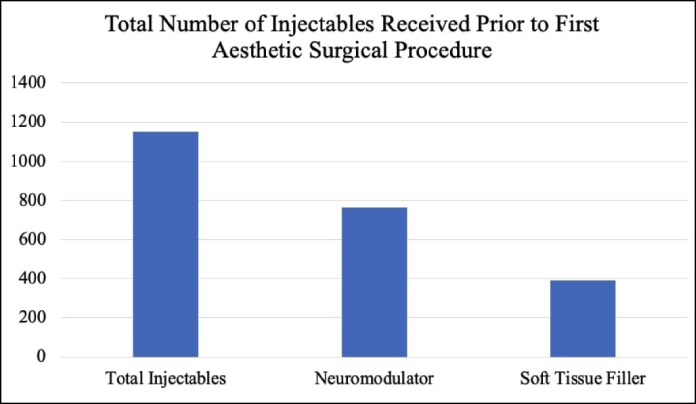
Total number of injectables received before the first aesthetic surgical procedure.

Of the 1368 patients who received botulinum toxin or soft-tissue fillers, 268 patients went on to undergo an aesthetic surgical procedure (19.6%). The most common procedure among these patients was a blepharoplasty (*n* = 149, 22.3%; Table 9). The average number of injectables received before the first aesthetic surgery performed was 4.3, with the average number of botulinum toxin injections being 2.9 and the average number of injectable soft-tissue fillers being 1.5 (Table 10, Figure 7). The median time period over which these injectable procedures were performed before the patients’ first aesthetic surgery was 325.5 days.

**Table 9.** Aesthetic Surgery Procedures Received After Injectables

Surgery type	*n*	%
Blepharoplasty	149	22.27
Rhytidectomy	123	18.39
Facial fat grafting	92	13.75
Breast augmentation	75	11.21
Mastopexy	51	7.62
Liposuction—trunk	40	5.98
Rhinoplasty	34	5.08
Liposuction—upper extremity	26	3.89
Abdominoplasty	20	2.99
Brow lift	15	2.24
Liposuction—head/neck	10	1.49
Body lift	9	1.35
Liposuction—lower extremity	9	1.35
Fat grafting—trunk	7	1.05
Brachioplasty	7	1.05
Breast reduction	2	0.30

**Table 10.** Average Number of Injectables Received Before First Aesthetic Surgical Procedure

	Mean	Standard deviation
Injectables	4.30	5.68
Neuromodulator	2.85	4.07
Soft-tissue filler	1.46	2.65

Eight patients (0.6%) received an aesthetic surgical procedure before receiving their first injectable from the investigator. Before receiving botulinum toxin or soft-tissue fillers, these patients received an average of 1.8 aesthetic surgical procedures. The most common surgery performed was facial fat grafting (*n* = 9, 64.3%; Table 11).

**Table 11.** Aesthetic Surgeries Before Injectables

Surgery type	*n*	%
Facial fat grafting	9	64.29
Rhytidectomy	1	7.14
Blepharoplasty	1	7.14
Liposuction—trunk	1	7.14
Liposuction—upper extremity	1	7.14
Fat grafting—trunk	1	7.14

## DISCUSSION

Injectables, such as botulinum toxin and soft-tissue fillers, are a versatile tool for addressing patients’ diverse aesthetic concerns. Their use has been associated with significant improvements in psychological and social functioning and reductions in appearance-related distress.^16,17^

### Botulinum Toxin

The popularity of neuromodulator injections has been steadily increasing. These treatments have consistently ranked as the Number 1 most commonly performed nonsurgical cosmetic procedure since 2005, according to the annual Aesthetic Society Cosmetic Surgery National Data Bank.^5,18-33^ These trends are consistent with those observed in this study (Figure 3). The decrease in 2020 can likely be attributed to the COVID-19 pandemic, especially given the rapid correction and continued increase in neuromodulator injection cases in the years following the pandemic.

Known adverse effects (AEs) of neurotoxin injection include local reactions (bleeding, pain, edema, erythema, and ecchymosis at the injection site), muscle weakness/paralysis (unintended local diffusion to neighboring muscle), systemic reactions (hypersensitivity reactions to BoNT or its components), and headaches.^34,35^ Cote et al reported that among 995 cosmetic cases with nonserious AEs, the most common were lack of effect (623 cases, 63%), injection-site reactions (190 cases, 19%), and ptosis (111 cases, 11%).^36^ Zargaran et al conducted a systemic review and meta-analysis of 4268 BoNT-A injection sessions and 1234 placebo cases and reported an overall complication rate of 16% with the breakdown as follows: headache and migraine (6.3%), local skin reactions (3.8%), facial neuromuscular symptoms (3.3%), pulmonary symptoms (2.1%), ocular symptoms (0.9%), cardiovascular symptoms (0.5%), gastrointestinal symptoms (0.4%), remote skin reactions (0.3%), face asymmetry (0.1%), and general symptoms such as fatigue (0.1%).^37^

Although there is an abundance of literature on adverse events following neuromodulator injections, few studies have examined complications over a 10-year period with each procedure performed by the same provider.^1,3,38^ In this study, ecchymosis (*n* = 10, 28.6%) and facial neuromuscular symptoms, such as overactivity/animation (*n* = 7, 20.0%), brow ptosis (*n* = 3, 8.6%), eyelid ptosis (*n* = 3, 8.6%), and asymmetry (*n* = 6, 17.1%), were the most notable complications following neurotoxin injections (Table 6).

**Figure 8.**
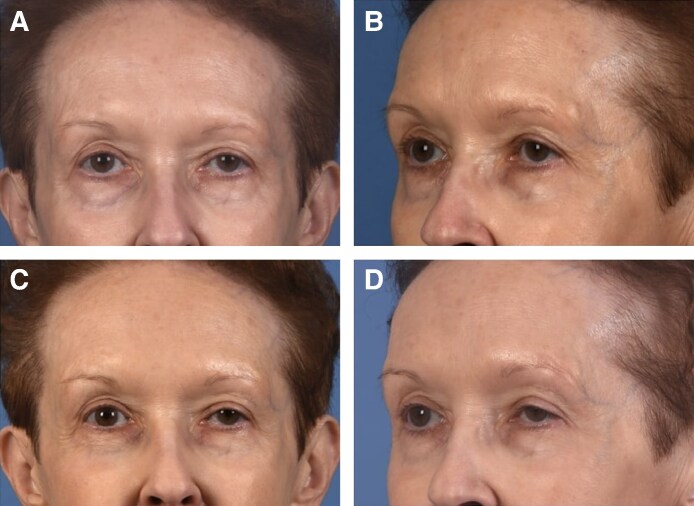
Case example: eyelid ptosis following botulinum toxin injection. The patient is a 72-year-old female presenting with an aging face. (A) Preprocedure photograph, frontal view. (B) Preprocedure photograph, left-sided view. (C) Postprocedure Day 21 photograph, frontal view. (D) Postprocedure Day 21 photograph, left-sided view.

**Figure 9.**
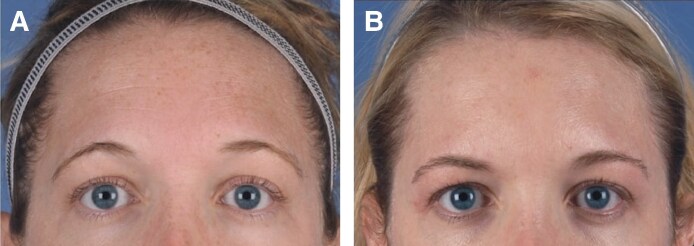
Case example: brow ptosis following botulinum toxin injection. The patient is a 36-year-old female presenting with an aging face. (A) Preprocedure photograph. (B) Postprocedure Day 24 photograph.

Local diffusion of product to neighboring muscles leading to iatrogenic blepharoptosis is a well-studied effect following neuromodulator injections.^10,39^ In this study, eyelid ptosis and brow ptosis occurred in 3 neurotoxin cases each (Figures 8, 9). Asymmetry occurred in 6 cases. One of these patients received neurotoxin injection to the mentalis and the platysma became affected, leading to an asymmetrical smile, which was addressed by injecting the contralateral side to improve symmetry. Another patient had forehead asymmetry, causing a “spock-like” appearance to the brow.

Epiphora, or excessive tearing from the eyes, is a rare but notable side effect of botulinum injections.^40^ One patient (2.9%) in this study developed epiphora bilaterally a few days after receiving Dysport injections to the forehead, lateral orbit, nasalis, pretarsal orbit area, and lower lids, and this was treated with alpha agonist drops.

Although the adverse events among the botulinum toxin products are similar, the risk may vary based on the product. Ahsanuddin et al analyzed the adverse event profile of Botox injections using the FDA Adverse Event Reporting System database.^41^ The top 5 adverse events were reported for each product. For Botox/Botox Cosmetic: ineffective drug (2.7%), headache (1.5%), nausea (1.0%), muscular weakness (1.0%), and fatigue (1.0%). For Dysport: dysphagia (2.6%), muscular weakness (2.2%), drug ineffectiveness (2.0%), fatigue (1.8%), and dyspnea (1.8%). For Xeomin (Merz, Raleigh, NC): drug ineffective (1.6%), headache (0.7%), injection-site pain (0.7%), therapeutic response decreased (0.7%), and dysphagia (0.6%). Notably, Dysport appears to have had the highest rate of muscular weakness (2.2%). Unlike Xeomin, the manufacturers do not recommend a 1:1 conversion for Dysport and instead recommend 50 U of Dysport for the equivalent of 20 U of Botox or Xeomin. This, accompanied with the “wider diffusion pattern” of Dysport, might explain the increased risk of side effects attributable to toxin diffusion especially when there is a small difference between targeted and untargeted muscles.^42^

### Soft-Tissue Fillers

Much like neuromodulator injections, the popularity of soft-tissue fillers has been steadily increasing. These treatments have consistently oscillated between the Number 2 and Number 3 most commonly performed nonsurgical cosmetic procedure since 2005 according to the annual Aesthetic Society Cosmetic Surgery National Data Bank.^5,18-33^ These trends are consistent with those observed in this study (Figure 3). Similar to our hypothesis for the decrease in neuromodulator injections in 2020, the decrease in soft-tissue fillers in 2020 can likely be attributed to the COVID-19 pandemic.

Known complications of soft-tissue fillers include local injection-site reactions, nodules, infections, vascular complications, and the Tyndall effect.^34^

**Figure 10.**
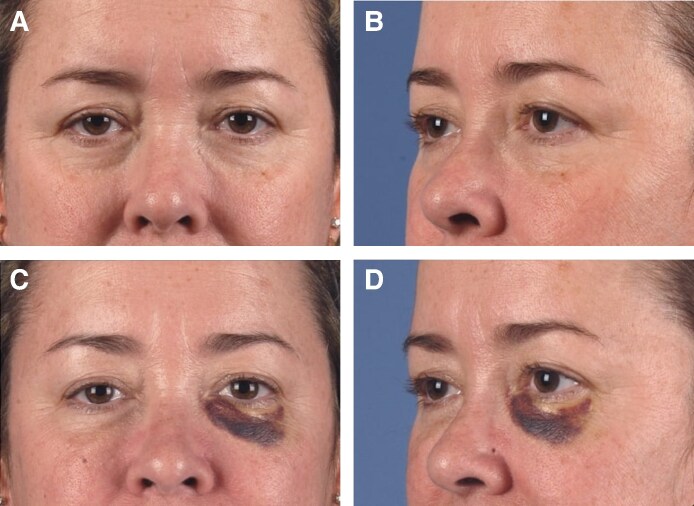
Case example: bruising following belotero filler injections of orbital rim, commissures, and nasolabial fold bilaterally. The patient is a 53-year-old female presenting with aging face. (A) Preprocedure photograph, frontal view. (B) Preprocedure photograph, left-sided view. (C) Postprocedure Day 3 photograph, frontal view. (D) Postprocedure Day 3 photograph, left-sided view.

Location injection-site reactions are most common and include swelling, bruising, pain, and pruritus (Figure 10). In this study, swelling related to inflammation occurred in 9 cases using hyaluronic acid products (Belotero, Restylane, Juvéderm) injected into the tear trough, orbital rim, or commissures. Local injection-site reactions are known to occur in 90.6% to 93.5% of patients following filler injections.^8,34,43,44^

**Figure 11.**
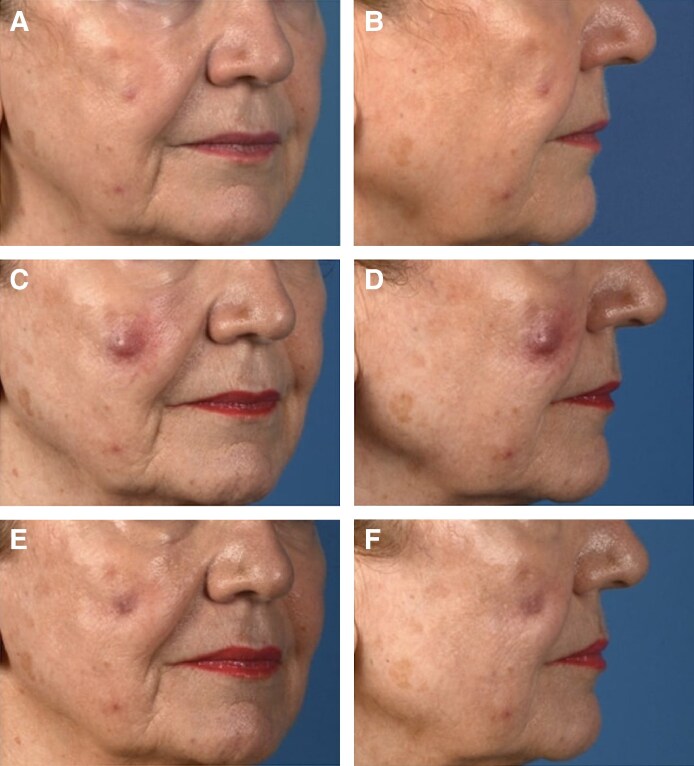
Case example: *Mycobacterium abscessus* infection following Voluma filler injections of the cheeks bilaterally. The patient is a 73-year-old female presenting with aging face. (A, B) Postprocedure Day 10 photographs. (C, D) Postprocedure Day 46 photographs. (E, F) Postprocedure Day 111 photographs.

Infection is a known complication of soft-tissue fillers that is well-described in the literature. All fillers, especially those that are longer lasting, can serve as a nidus for infection and biofilm formation.^44^ In this study, there were 3 cases of infection, 2 of which were because of *Mycobacterium* species and 1 of which was because of *Staphylococcus* species (Figure 11). At the time, the soft-tissue filler procedures were done using ice placed directly on the skin to provide analgesia. After the *Mycobacterium* infections were identified, it was found that the ice machine was contaminated. Following identification of this source, the technique changed and direct ice on the skin was no longer used, eliminating this potential risk.

Nodule formation is a well-known complication and occurred in 4 soft-tissue filler cases in this study and may be because of superficial placement of filler.^38,45,46^ In all 4 cases, nodule formation was localized and palpable. One patient who received hyaluronic acid injection to the orbital rim and tear trough developed a nodular area on the right lateral cheek. This was treated with a series of hyaluronidase injections (60, 75, 50, and 20 units spaced 2-5 days apart).^46^ Two patients had upper lip nodules following hyaluronic acid injections for lip augmentation, for which massage was the recommended treatment.

Of note, Juvéderm was used in 75% of the cases that were complicated by nodule formation. In a study of 7659 patients receiving hyaluronic acid, calcium hydroxyapatite, or poly-L-lactic acid filler, the 3 reported cases of delayed nodule development all occurred in patients receiving either Juvéderm Voluma or Juvéderm Volbella.^47^ This increased risk of delayed nodules may be because of the ability of the low molecular weight hyaluronic acid chain in Juvéderm to activate the immune system.^47^

Palpability is the accumulation of filler that can be felt, although it may not be visible. In this study, palpability of one of the oral commissures following injection occurred in 3 cases (Juvéderm Volbella *n* = 2, Restylane Lyft *n* = 1). The appearance of lumps or bumps may cause asymmetry, which we noted in 4 cases, all of which involved injections to the cheeks/malar area and 3 of which also included the lips.

Recommendations to mitigate such AEs include proper injection techniques executed by trained healthcare providers, thorough anatomical evaluation, the application of cold compresses within the first 24 h postinjection, immediate postinjection massage, thorough patient education, avoidance of blood thinners, and diligent monitoring through follow-up visits.^45^ Although soft-tissue filler injections have a good safety profile, awareness and understanding of the potential complications is critical.

### Surgical Conversion Rates

Matarasso et al have hypothesized that patients who receive neuromodulator injections and soft-tissue fillers may undergo additional facial rejuvenation and antiaging therapies in the future; however, there is currently sparse literature quantifying any bidirectional overlap between patients receiving injectables and those receiving aesthetic surgery.^13^ In a retrospective cross-sectional study by de Almeida et al, 15.5% of patients in the per-protocol population who were treated with onabotulinumtoxinA (*n* = 194) underwent concomitant plastic surgery.^48^ Similarly, a retrospective study by Richards et al revealed that 15.7% of patients in the study's plastic surgery practice who received Botox or soft-tissue fillers subsequently underwent an aesthetic plastic surgery procedure.^14^ However, these studies did not report how many patients received aesthetic surgery before injectables. D’Amico et al described the 747 effect, wherein only 7% of patients with no previous history of cosmetic procedures would choose to go to a nonplastic surgeon for an invasive procedure.^15^ However, this percentage jumps to 47% for patients who previously had a positive experience with a nonplastic surgeon for a noninvasive procedure.^15^ Thus, these studies underscore the importance of neuromodulator injections and soft-tissue fillers in a plastic surgery practice, not only to have the option to offer these noninvasive treatments to patients but also to retain patients for possible future aesthetic surgical procedures.

In this study, the percentage of patients who received aesthetic surgery following injectables (19.6%) was larger than the percentage of patients who received injectables following aesthetic surgery (0.6%). This is likely because of the minimally invasive nature of injectables, which makes them a logical first therapeutic option before surgery. For this reason, many patients may initially desire Botox and/or soft-tissue fillers. However, the large percentage of patients who went on to receive surgery after injectables suggests that injectables may serve as a starting point for patients pursuing antiaging treatments and an opportunity to establish continuity of care for providers.

The top 3 surgeries received by both patients who had injectables first and patients who had injectables after were blepharoplasty, rhytidectomy, and facial fat grafting (Tables 8, 10). Patients who received injectables first had an average of 4.3 injectables before their first aesthetic surgery (Table 9). This suggests that early investment in injectables may prolong the need for an eventual facelift to treat facial aging.

### Limitations

This study has several limitations. Given its retrospective nature, it relies on the accuracy of the EMR, which may not capture all complications or nuances of each procedure. Furthermore, although this data includes 10 years’ worth of clinical data, it may be capturing shorter bursts of clinical engagement as opposed to a continuum of care lasting 10 years for each patient. Complications, such as significant bruising, brow ptosis, and incomplete correction, are likely underreported. Additionally, nomenclature of the injection sites as recorded in the EMR may be another limitation, because some patients received injections in the alar base but were included in the nasolabial fold group. This study does not include a control group, making it difficult to compare outcomes against other techniques or providers. Patient self-reporting and follow-up consistency could introduce bias or variability in the recorded outcomes. The study population consists primarily of Caucasian females, which may limit the generalizability of the findings to more diverse populations. Additionally, the findings described in this study may be influenced by the specialization of the senior author's own practice. For example, the focus on the senior author's practice on facial rejuvenation may have played a role in subsequent procedures that their patients chose to undergo. Lastly, the senior author individualized variations in technique, product preference, and injection sites per patient, which may limit reproducibility of the results by other practitioners.

## CONCLUSIONS

As plastic surgeons have been increasingly performing botulinum toxin and soft-tissue fillers, it is vital that key injection sites, common complications, and patient and provider preferences be examined. Additionally, because these procedures rise in popularity, it is important to understand how patients may transition from receiving injectables to aesthetic surgery or vice versa. The results of this study demonstrate that botulinum toxin and soft-tissue fillers are safe with low complication rates when administered by an experienced provider and that these procedures serve as a gateway to future aesthetic procedures. Given the sizeable conversion between patients receiving injectables to aesthetic surgical procedures and previous research demonstrating patient preferences for invasive cosmetic procedures following noninvasive procedures, injectables are a key component of plastic surgery practices.^15^
